# Incidence and evolution of nasal polyps in children and adolescents with cystic fibrosis

**DOI:** 10.1016/S1808-8694(15)30745-X

**Published:** 2015-10-19

**Authors:** Silke Anna Thereza Weber, Giesela Fleischer Ferrari

**Affiliations:** 1 PhD. Professor of Otolaryngology - Medical School of Botucatu; 2 PhD. Professor of Pneumopediatrics - Medical School of Botucatu

**Keywords:** diagnosis, endoscopy, cystic fibrosis, polyposis, therapy

## Abstract

Nasal polyps are a clinical sign of alert for investigating Cystic Fibrosis (CF).

**Aims:**

To study the incidence of nasal polyps in children and adolescents with cystic fibrosis, its possible association with age, gender, clinical manifestations, genotype and sweat chlorine level, and its evolution with topical steroid therapy.

**Methods:**

Clinical symptoms, sweat chlorine level and genotype were studied in 23 cystic fibrosis patients. Nasal polyps were diagnosed by nasal endoscopy and treated with topical steroids during 6 months, followed by a second nasal endoscopy. Fisher test was used for statistical analysis.

**Results:**

Nasal polyps were found in 39.1% of the patients (five bilateral, four unilateral), all older than six years, recurrent pneumonia in 82.6%, pancreatic insufficiency in 87% and malnutrition in 74%. No association was seen between nasal polyps and sweat chlorine level, genotype, clinical sings of severity and nasal symptoms. Seven patients improved in their nasal polyps with topical steroids, six showed complete resolution.

**Conclusion:**

The study showed a high incidence of nasal polyps in older children, who span the entire range of clinical severity, even in the absence of clinical nasal symptoms. Topical steroid therapy showed good results. An interaction among pediatricians and otolaryngologists is necessary for diagnosis and follow-up.

## INTRODUCTION

Cystic fibrosis (CF) is the most common lethal genetic disease in Caucasians, of autosomal, recessive transmission, at a rate of 1:2000 live births in this population^1^^,^^2^, in Brazil, the incidence is of 1:9500 in Paraná^3^, 1:8700 in Santa Catarina^4^ and 1:10.0005 in Minas Gerais.

The variable severity associated with the clinical manifestations (distinctive phenotypes) depends partially on the genotype and results from the obstructive phenomena, thus characterizing the cystic fibrosis:

1 Chronic suppurative obstructive pulmonary disease;
2 Pancreatic insufficiency with digestion problems and malabsorption, resulting in secondary malnutrition;
3 Increased concentrations of chlorine and sodium, and age.
4 Adult male infertility.

Symptoms onset varies broadly, depending on mutation type, homozygote patients for the genetic mutation 5F508 start having symptoms in the first 2–4 months of life. The classic clinical picture starts with dry cough, tachypnea, mild intercostal pulling, or, it may manifest itself as acute infection, like bronchiolitis. The clinical course evolves with recurrent pneumonia. Together with all of this, the patient has difficulty gaining weight, despite a voracious appetite, enlarged and more frequent foul-smelling defecation, diarrhea or steatorrhea (oily feces)^12^.

Cystic fibrosis is diagnosed based on at least two of the four clinical-laboratorial aspects: family history of cystic fibrosis, pancreatic insufficiency, chronic suppurative obstructive pulmonary disease and high levels of chlorine and sodium (>60mEq/l) in their sweat secretion. Other clinical data that suggest the diagnoses are: meconium ileum and/or intestinal obstruction with atresia, deficient weight-height development, heat stress, chronic pansinusitis, nasal polyps, volvus and intusception, and azoospermia^6^^,^^7^.

Clinical manifestations in the upper airways (UAW) happen to 100% of the patients, including recurrent sinusitis, rhinitis and/or nasal polyposis^8^, ^9^, ^10^, ^11^. The incidence of nasal polyps has been reported in 6 to 48% of the cases^12^^,^^13^, by the time cystic fibrosis is diagnosed, about 4% of the patients have some symptoms associated with nasal polyps. It is believed that about 14% of the patients with cystic fibrosis will require surgery to treat the polyps^8^^,^^10^^,^^11^^,^^14^.

Based on these data from the literature, the departments of pediatric pneumology and otorhinolaryngology of the Botucatu Medical School - UNESP, decided to assess UAW involvement in patients with cystic fibrosis in the outpatient ward.

## OBJECTIVE

The general goal of our paper was to assess nasal polyp incidence through endoscopy in children and adolescents with cystic fibrosis being followed in the outpatient ward. The specific goals were:

1- to assess age, gender, clinical symptoms and the genetic mutation of these patients, and the association between these data and nasal polyposis;
2- assess polyposis evolution with topical steroids.

## PATIENTS AND METHODS

The present contemporary cross-sectional and prospective cohort was approved by the Ethics in Research Committee of the Botucatu Medical School - UNESP, under protocol # 1743/2005. The parents/guardians signed an Informed Consent Form.

In 2005 we assessed the 23 patients being followed at the Cystic Fibrosis Reference Center Outpatient Ward, with ages ranging between 1 year and 9 months and 22 years and 8 months.

From their charts, we obtained data related to gender, age, clinical manifestations of CF such as meconium ileum, malnutrition, pancreatic insufficiency and repetition pneumonia, and laboratorial exams to confirm CF, such as quantitative analysis of ion content in sweat^7^ and genetic studies. All patients underwent otolaryngological evaluation and suffered nasal endoscopy. During the consultation we obtained information related to nasal obstruction, oral breathing, asthma and sinusitis.

Nasal endoscopy was carried out under topical anesthesia, using a flexible Storz pediatric bronchoscope of 2.4mm in diameter, or a rigid 30°, 2.4mm Storz scope.

In the nasal exam we described whether or not polyps were present, following the staging classification proposed by Johansson et al.^15^ (level 0 - absent, level I - polyp in the middle meatus, level II - polyp going through the middle turbinate with clear nasal floor, level III - polyp filling up the entire nasal cavity, whether or not there is secretion and its color, nasal mucosa aspect (color, edema, degeneration).

Those patients with nasal polyposis were prospectively followed up and submitted to clinical treatment with topical steroids in the habitual dose (mometasone 200 mcg per day) for 6 months. After this period, the nasal endoscopic exam was repeated.

For statistical analysis, the data obtained were described in their mean and standard deviation values. Age, gender, clinical symptoms and genetic mutations were associated with the presence of polyps. We used Fisher's Exact Test, at a significance level of p<0.05.

## RESULTS

The median age of the 23 patients was of six years and four months, and 20 of them were males.

Most of the patients presented with the classical clinical manifestations, and 82.6% had repetition pneumonia, 87% had pancreatic insufficiency, 74% malnutrition and 13%, meconium ileum. CF was confirmed in all of them, with too high chlorine levels in the sweat, the mean value was of 92.093 ± 24.625 mEq/l. Genetic mutations were investigated in all subjects. We found eight patients with 5F 508/other genetic mutation, three 5F 508/5F508, one 5F508/G 542X, one G542X/other, one R1162X/R1162X and in nine patients we did not observe mutations.

As to respiratory complaints, 35% of the patients had asthma, 22% had frequent sinusitis - as diagnosed by pediatric pneumologists and 22% were diagnosed with oral breathing.

**Figure 1:**
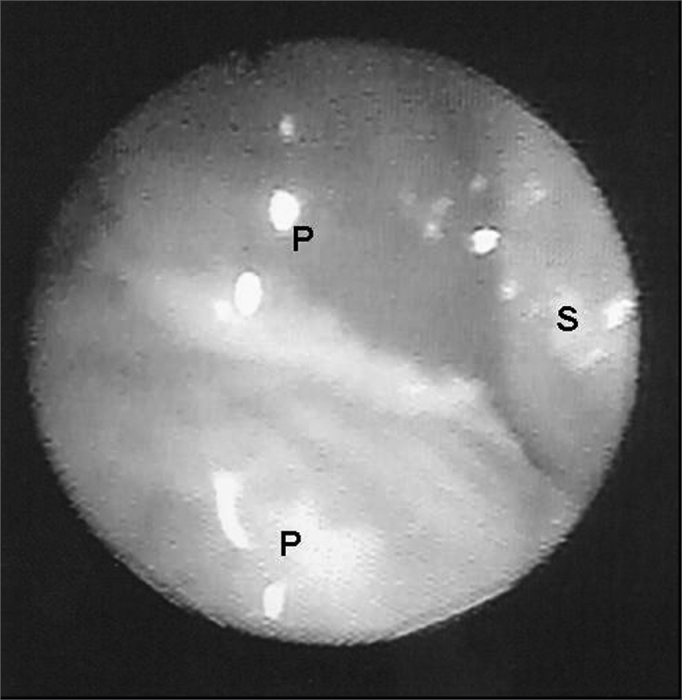
Endoscopic image of a grade III polyp in the right nasal cavity of patient #1. P - polyp S - nasal septum

**Figure 2:**
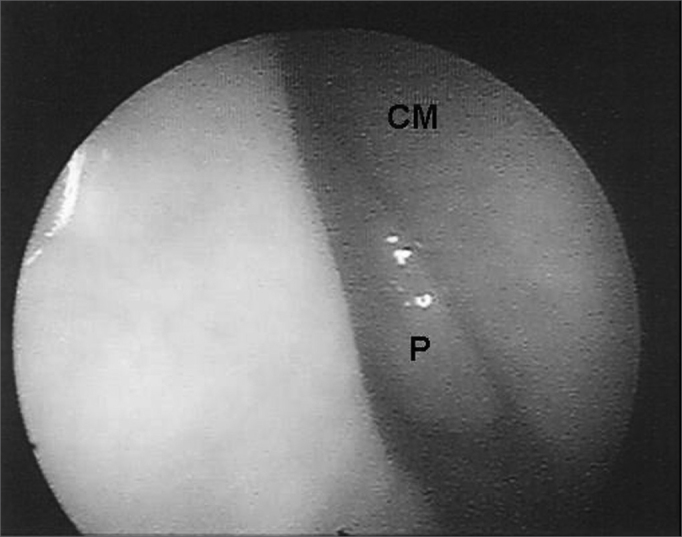
Endoscopic image of a grade I nasal polyp in the right nasal cavity of patient # 7. - P - polyp CM - middle turbinate

**Graph 1:**
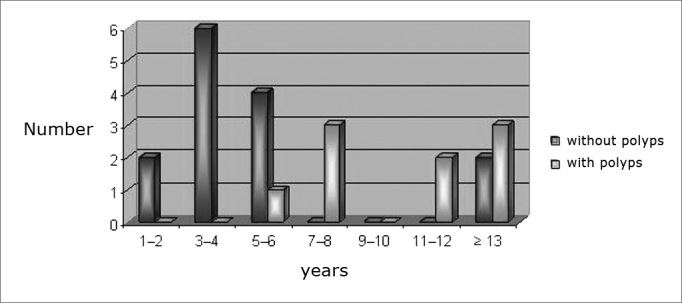
Distribution of cystic fibrosis patients in relation to age and nasal polyps present.

Nasal endoscopy revealed nasal polyps in 39.1% (nine patients) of the patients. Of these, five patients had bilateral polyposis and four had unilateral disease, level I in four, level II in one patient and Level III in four patients (Figure 1, Figure 2). Nasal polyposis was diagnosed in children as of six years and three months of age, with incidence increase as age increased (Graph 1). There was no association between gender, age, clinical severity and genetic mutation with nasal polyposis.

After six months of clinical treatment with topical steroids in the habitual dose, we observed that of the nine children with polyps, seven (67.7%) improved. Six had complete polyp remission. Of these, in regards to the classification, two had bilateral level III polyposis, one had bilateral level II polyposis and three had unilateral level I polyps. One patient had his polyposis improve from level III to level II. Two patients with bilateral level III and another with unilateral level I did not show any improvement. One patient had level I unilateral polyp involution, however he later had a new level I polyp in the contralateral side - according to the endoscopic exam (Table I).

**Table 1:** Clinical manifestations in patients with cystic fibrosis and nasal polyps and its evolution with clinical treatment.

Patient	Age(m)	Asthma	Sinusitis	Oral Breathing	Genetic mutation	Polyposis and Treatment	
						Before	After
01	103	No	No	No	R1162X/R1162X	Bilateral Grade III	Bilateral Grade III
02	102	Yes	No	No	▴F508/?	Right Grade I	Absent
03	139	Yes	No	No	?/?	Left Grade I	Left Grade I
04	75	No	No	No	G542X/?	Bilateral Grade III	Absent
05	106	Yes	Yes	Yes	?/?	Bilateral Grade III	Absent
06	139	No	No	No	▴F508/?	Bilateral Grade III	Bilateral Grade II
07	272	No	No	No	?/?	Right Grade I	Right Grade I
08	179	Yes	No	No	?/?	Bilateral Grade III	Absent
09	164	Yes	No	No	?/?	Left Grade I	Absent

## DISCUSSION

The patients assessed during diagnosis had the classical cystic fibrosis clinical signs and symptoms, such as meconium ileum, pancreatic insufficiency, malnutrition and repetition pneumonia. Diagnostic confirmation was carried out based on two abnormal levels of chlorine ion in their sweat.

Sinusal disease, diagnosed by CT scan, has been reported in CF in up to 100% of the cases^9^^,^^10^^,^^11^^,^^13^^,^^14^, although the percentage of adult patients with clinical manifestation is much lower^7^^,^^8^^,^^13^. It is believed that the thick mucus reduces ciliary movement, causes meatal obstruction and both hypercapnia and hypoxia facilitate the development of local infection/inflammation. In the population studied we observed an incidence of sinusitis and oral breathing in 22% of the patients, and such incidence rates are similar to the ones reported by other authors^9^^,^^10^^,^^13^^,^^19^; however only one of these patients reported nasal polyps. Similar data were published in 2002 by Henriksson et al.^13^ in a study involving 111 patients with cystic fibrosis with median age of 18 years. During nasal endoscopic exam they observed nasal polyps in 39% of the patients; however they found no correlation between nasal obstruction, nasal secretion and polyps in these patients.

In the literature, nasal polyposis has been reported at an incidence rate of 6 to 48% in patients with CF^10^^,^^11^^,^^17^, and a recent national study16 showed that children of a median age of 9.5 years had an incidence rate of 15.2%. In the present study, the incidence of nasal polyposis was similar to the one reported in the literature; however, consider the finding of 39.1% of children and adolescents with high levels of polyposis, because the aforementioned studies included adults in their series. When patients below 10 years of age were assessed, the incidence reported was between 5 and 15.2%^16^^,^^18^. Schmitt et al.^18^ in 2005, assessing 893 children with CF, found nasal polyps in only 5% of the children, and in 0.2% cystic fibrosis was diagnosed due to the presence of polyps. This is a very important piece of data, because although the isolated polyp does not always mean cystic fibrosis, the otolaryngologist must investigate it because of its severity and importance in early diagnosis and treatment in order to provide a better survival prognosis.

In 1992, Ramsey et al.^11^ had already seen small polyps during endoscopic exam, unseen by anterior rhinoscopy. Similar to the study carried out by Henriksson et al.^13^, the polyps were classified as small in 68% of the patients; in 23% the polyps were in the meatus, being diagnosed only by means of nasal endoscopy. In our study, four (45%) patients had small polyps, stressing the importance of routine nasal endoscopy and clinician-otolaryngologist interaction.

It is believed that up to 20% of the patients with nasal polyps require surgery^8^^,^^9^^,^^11^, and endoscopic surgery is the best approach8,20. According to Kingdom et al.^14^, the 5F 508 genetic mutation is the most relevant in patients with CF and polyposis, requiring nasal surgery. In our study, we did not find any association between nasal polyp presence or severity and genotype.

Surgical recurrences are frequent, reported in up to 13% of the patients who undergo functional surgery. Thus, there are authors who suggest treatment with topical steroids as a routine approach to treat nasal polyps, resorting to surgery only in cases of failures^21^. In the group studied, topical steroids showed improvement in seven (77.7%) patients, of whom 85.7% had complete polyp involution. These results are better than the ones seen in the literature, showing polyposis improvement in 56% of the cases8. For the specific population of patients with cystic fibrosis there is no data about clinical treatment development in longer periods.

## CONCLUSIONS

In the population studied, the incidence of nasal polyps was high (39.1%), and was diagnosed in children above six years of age. There was no association between nasal polyps and age, gender, genetic mutation, chlorine levels in sweat or clinical symptoms. Polyposis treatment with steroids proved efficacious in 77.7% of the patients.

## FINAL REMARKS

Nasal polyps in patients with cystic fibrosis are common, even in the pediatric population, unrelated with nasal symptoms. Its diagnosis must be endoscopic. It is fundamental to have pediatric pneumologists working closely with otolaryngologists for better diagnosis and follow up.
